# Calculating Orthologs in Bacteria and Archaea: A Divide and Conquer Approach

**DOI:** 10.1371/journal.pone.0028388

**Published:** 2011-12-12

**Authors:** Mihail R. Halachev, Nicholas J. Loman, Mark J. Pallen

**Affiliations:** School of Biosciences, University of Birmingham, Birmingham, United Kingdom; J. Craig Venter Institute, United States of America

## Abstract

Among proteins, orthologs are defined as those that are derived by vertical descent from a single progenitor in the last common ancestor of their host organisms. Our goal is to compute a complete set of protein orthologs derived from all currently available complete bacterial and archaeal genomes. Traditional approaches typically rely on all-against-all BLAST searching which is prohibitively expensive in terms of hardware requirements or computational time (requiring an estimated 18 months or more on a typical server). Here, we present xBASE-Orth, a system for ongoing ortholog annotation, which applies a “divide and conquer” approach and adopts a pragmatic scheme that trades accuracy for speed. Starting at species level, xBASE-Orth carefully constructs and uses pan-genomes as proxies for the full collections of coding sequences at each level as it progressively climbs the taxonomic tree using the previously computed data. This leads to a significant decrease in the number of alignments that need to be performed, which translates into faster computation, making ortholog computation possible on a global scale. Using xBASE-Orth, we analyzed an NCBI collection of 1,288 bacterial and 94 archaeal complete genomes with more than 4 million coding sequences in 5 weeks and predicted more than 700 million ortholog pairs, clustered in 175,531 orthologous groups. We have also identified sets of highly conserved bacterial and archaeal orthologs and in so doing have highlighted anomalies in genome annotation and in the proposed composition of the minimal bacterial genome. In summary, our approach allows for scalable and efficient computation of the bacterial and archaeal ortholog annotations. In addition, due to its hierarchical nature, it is suitable for incorporating novel complete genomes and alternative genome annotations. The computed ortholog data and a continuously evolving set of applications based on it are integrated in the xBASE database, available at http://www.xbase.ac.uk/.

## Introduction

A central goal in comparative genomics is to identify novel and/or shared biology between organisms, or at least make informed predictions in this regard. The discovery of sets of orthologous proteins plays an important role towards elucidating such relationships. Fitch [1] originally proposed the definition of orthologs as homologous proteins related via speciation. Under this definition of orthologs “it is both theoretically plausible and empirically supported that due to their sequence similarity they have similar structure and typically perform equivalent biological function” [2]. Paralogs, on the other hand, are created via gene duplication and are prone to diversification, which can lead to them acquiring biologically distinct functions. As lines of descent are rarely known, a practical approach for inferring orthology is to compare protein sequences and draw conclusions based on sequence similarity. The existence of co-orthologs, i.e. where a pair of paralogs from one genome is orthologous to a protein or a pair of paralogs from another, can complicate such approaches and requires further consideration.

Assignments of orthology are required in numerous contexts, including determining gene content and creating annotation for newly sequenced genomes; taxonomic and phylogenetic studies; estimation of the number of novel genes expected when sequencing a new strain from a known species [3]; and identifying novel drug targets [4].

Existing techniques for ortholog computation fall in two major categories – tree-based and pair-based. Tree-based techniques (e.g., [5]–[10]) identify all similar genes among a set of genomes, build a phylogenetic tree for each family of homologs and use the trees to distinguish between orthologs and paralogs (orthology inferred if tree for protein identical to that for whole genomes). Pair-based techniques (e.g., [11]–[25]) use a heuristic approach to identify pairs of similar genes belonging to different genomes and then organize them into orthologous groups by performing a subsequent clustering step to filter out some co-orthologous pairs.

Tree-based techniques are generally thought to perform better than pair-based approaches [26], but in many cases it appears that the two approaches perform equally well [27], [28]. Even if considered the “gold standard”, tree-based approaches are not feasible for large projects, involving millions of proteins, as they are slow and resource-hungry in computational terms and difficult to automate. OrthoMCL is a popular representative of the pair-based approach to ortholog computation, providing an attractive trade-off between sensitivity and specificity [27], [28] and ability to handle evolutionary distant sequences. In addition, OrthoMCL analyses are not limited to pair-wise comparisons and can be automated.

We wished to compute orthologs for the large and ever-increasing set of complete prokaryotic genomes. However, an off-the-shelf adoption of OrthoMCL for analyzing the current 1,382 complete prokaryotic genomes from the NCBI collection would make unrealistic demands on time or computational capacity. Based on the OrthoMCL algorithmic complexity – a quadratic function of the number of coding sequences (CDSs) alignments to be performed, we estimate that it would take more than 18 months to analyze the current 1,382 genomes on a typical server, i.e. four 2.3 GHz CPUs and 16 GB RAM (see Results). In other words, if we were to start the ortholog computation using directly OrthoMCL today, by the time the computation was finished, we would be faced with a four-fold bigger problem, given the NCBI's 18-month doubling rate for prokaryotic genomes and the solution's quadratic complexity. One might argue that such a problem could still be solved in reasonable time given extensive use of computational resources (e.g. in the cloud). However, even this solution is not sustainable, as three years from today the problem will be 16 times larger and, in only nine years, it will have increased over 4,000-fold!

Faced with these challenges, we devised an alternative approach, xBASE-Orth, to provide a scalable and efficient system for ortholog annotation. xBASE-Orth is a pair-based technique, which relies on bidirectional best-hit calculations, applies a “divide and conquer” approach and adopts a pragmatic scheme that trades accuracy for speed. Starting at species level, xBASE-Orth carefully constructs and uses pan-genomes as proxies for the full collections of coding sequences at each level as it progressively climbs the taxonomic tree using the previously computed data (see Methods). This leads to a significant decrease in the number of alignments that need to be performed, which translates into faster computation, making ortholog computation possible on a global scale.

## Results

### Predicted Ortholog Pairs and Groups

**Figure 1:**
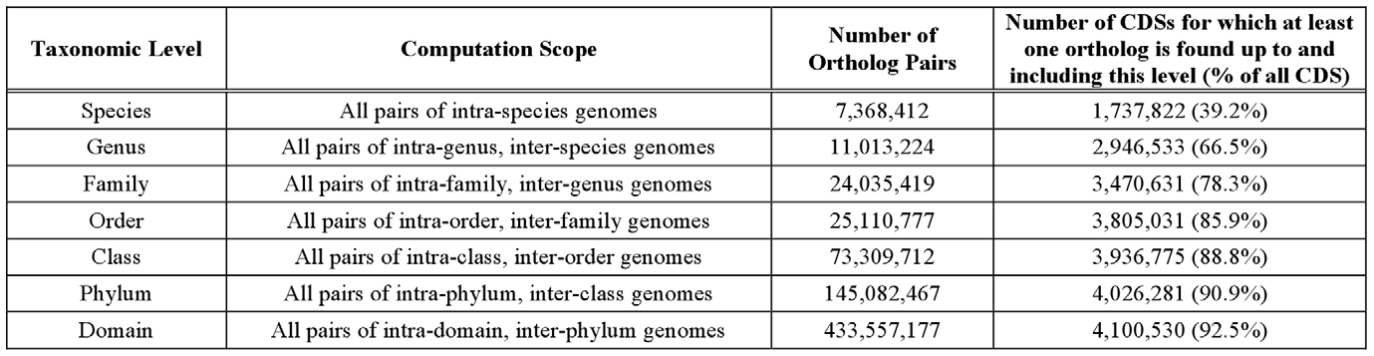
Discovered Ortholog Pairs (OP) analyzing 1,382 complete prokaryotic genomes. xBASE-Orth computes the ortholog pairs by climbing up taxonomic levels and using the results at lower levels as part of the input for higher levels. At species level, for each species with two or more complete genomes, we compute the orthologs for each pair of genomes (see Methods). At genus level, for each genus with two or more species with at least one complete genome, we compute the orthologs for each inter-species genome pair. For details on how the species ortholog data is used in ortholog computation at genus level, see Methods. The orthologue computation at higher levels proceeds similarly.

Using xBASE-Orth, we analyzed 94 archaeal and 1,288 bacterial complete genomes available in the NCBI's RefSeq collection (February 2011, see Dataset S1) containing 4,431,241 CDSs. As can be seen from Figure 1, we found a total of 719,477,188 ortholog pairs analyzing all possible genome pairs in the bacterial and archaeal domains. xBASE-Orth could not find any ortholog for 7.5% of the CDSs; for the remaining 92.5% of the CDSs there are ∼350 orthologs per CDS on average.

**Figure 2:**
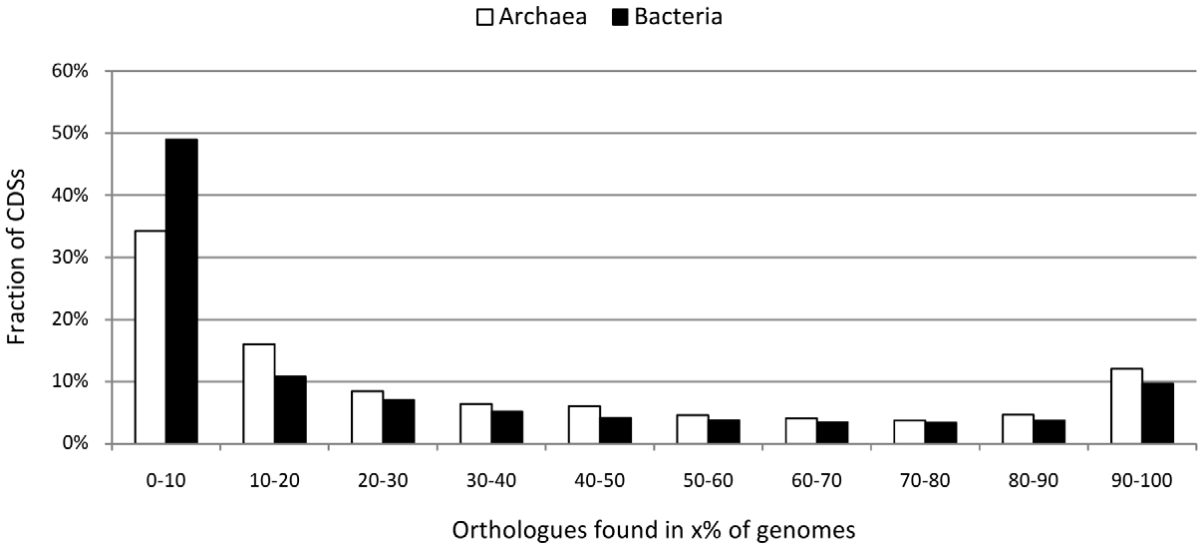
Frequency of CDSs occurrence in the 94 archaeal and 1,288 bacterial genomes. Using single-linkage approach, the computed orthologs are organized in ortholog groups (OGs). Each CDS is clustered to an OG if it forms an ortholog pair with at least one CDS from the group and each CDS is included in one OG only. The x-axis lists 10 possible bins for the observed percentage of genomes in which CDSs from a particular OG are found. The y-axis denotes the fraction of all CDSs found for each bin. In agreement with previous reports, a significant fraction of CDSs are found only in a small percentage of genomes (less than in 10% of the considered archaeal or bacterial genomes, these also include singletons/orphans) forming the “accessory pool”; about 10% of the CDSs have orthologs in more than 90% of the genomes - the “extended core”.

Using the ortholog pairs data, we organized the CDSs in ortholog groups (OGs) using the single-linkage approach (i.e., a CDS is clustered to an ortholog group if it forms an orthologous pair with at least one CDS from this group). Each of the generated 15,874 (Archaea) and 159,657 (Bacteria) ortholog groups contains 2 or more CDSs and each CDS belongs to one group only. Based on the OGs, we computed the frequency of occurrence of the CDSs in the considered genomes (Figure 2). Our results are in accordance with the estimations made in [29]: a significant fraction of all CDSs (about one-third for Archaea and one-half for Bacteria in our computation) are present only in a small percentage of genomes (less than or equal to 10%) – i.e. the “accessory pool”; about 10% of all CDSs have orthologs in more than 90% of the genomes – i.e. the “extended core”.

The computed orthologs are integrated in the xBASE database (http://xbase.ac.uk/). The ortholog pairs/ortholog group for a CDS can be fetched via the xBASE web-interface by entering the CDS locus tag in the search box, following the “Genome View” link, and selecting the desired option from the left-hand panel.

### Timing and Speedup

**Figure 3:**
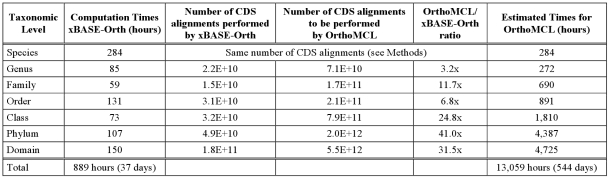
Computation times and estimated speedup compared to OrthoMCL. By adopting practical accuracy/speed trade-off xBASE-Orth allows for acceptable computational time using reasonable hardware resources and is predicted to be about 15 times faster compared to direct application of OrthoMCL. The vast majority of the computation time is spent performing CDS alignments and operating on the smaller pan-genomes (xBASE-Orth) rather than on the full CDS collection (OrthoMCL) results in significant time advantage. It is more prominent at higher taxonomic levels, where the difference between pan-genome and full CDS collection sizes increases.

We implemented the xBASE-Orth pipeline in Python and conducted the experiments using a 4×2.3 GHz CPUs, 16 GB RAM Blade server. Analyzing the 1,382 complete prokaryotic genomes took a total of 889 hours (∼37 days), the vast majority of which (>80%) was spent performing alignments of amino acid sequences. Figure 3 shows a more detailed report on the computation times for xBASE-Orth at each level, as well as the estimated speedup achieved by xBASE-Orth compared to a brute-force use of OrthoMCL. The estimation of the OrthoMCL computation times is based on the fact that both xBASE-Orth and OrthoMCL computation times are quadratic functions with respect to the number of CDSs to be aligned. Since xBASE-Orth uses pan-genomes rather than the full CDS collections (see Methods), it performs an order of magnitude fewer alignments (Columns 3 and 4), resulting in estimated overall speedup of about 15-fold.

### Comparison with OrthoMCL

**Figure 4:**
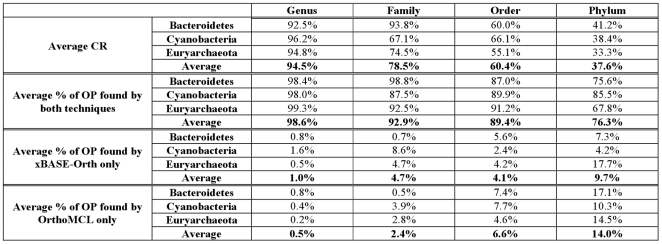
xBASE-Orth vs. OrthoMCL comparison. xBASE-Orth has a significant speed advantage over direct application of OrthoMCL which comes at the possible cost of decreased accuracy. We compared the performance of the two approaches over three distinct phyla – Bacteroidetes (40 complete genomes), Cyanobacteria (41 genomes) and Euryarchaeota (62 genomes), computing orthologs at genus, family, order and phylum level. At higher taxonomic levels the pan-genome sizes are significantly smaller compared to the full CDS collections - about half at order level and only about one third at phylum level for the datasets analyzed here (CR stands for Compression Ratio = [Number of CDSs in pan-genomes used by xBASE-Orth/Number of CDSs used by OrthoMCL] * 100%). Hence, at higher levels each CDS in a pan-genome is a representative of a larger set of orthologous/paralogous CDSs, plausibly becoming less sensitive and specific. Compared to the OrthoMCL results, it appears that on average the xBASE-Orth results contain from 1% (at genus level) to 9.7% (phylum) additional ortholog pairs, while failing to detect from 0.5% (genus) to 14% (phylum) OrthoMCL pairs.

To evaluate the accuracy of xBASE-Orth, we compared its predicted ortholog pairs (OP) to the OP produced by OrthoMCL (given the full set of CDSs) for three evolutionary distant phyla – Bacteroidetes (40 complete genomes), Cyanobacteria (41 genomes) and Euryarchaeota (62 genomes). Using pan-genomes as proxies to the full CDS collections provides a significant computation speedup for xBASE-Orth, but introduces some differences in the predicted orthologs (Figure 4). The difference in the predicted ortholog pairs between the two techniques increases with the evolutionary distance. At higher taxonomic levels the pan-genomes are more compressed, i.e. each CDS in a pan-genome is a representative of a larger set of orthologous/paralogous CDSs, thus becoming less sensitive and specific.

### xBase-Orth Sensitivity and Specificity

To determine the quality of the xBASE-Orth ortholog prediction, we investigated its sensitivity and specificity. In our context, sensitivity refers to the ability to discover distant orthologs. As shown in Figure 1, xBASE-Orth could not find orthologs for 7.5% of the CDSs, ∼80% of which are annotated as “hypothetical proteins”. This number is in accordance with the results reported previously for detecting singleton ORFans (i.e. CDSs with no homology with any other protein from a genome collection) - 14.4% analyzing 127 microbial genomes [30], 14% in 60 genomes [31], 12% in 122 genomes [32], 7.8% in 277 genomes [33] and typically 10–15% [34]. For the remaining 92.5% of the CDSs, the average distribution of orthologs found across the taxonomic levels is: 2% at species and genus level each, 4% at family and order level each, 12% at class level, 21% at phylum level and 55% at domain level, which indicates suitable sensitivity of xBASE-Orth.

To investigate the specificity of xBASE-Orth, i.e. avoiding spurious ortholog pairs, we performed the following analysis. For a given CDS, we fetched all of its predicted orthologs, performed a multiple alignment of the CDSs in this orthologous group with ClustalW2 [35] using default parameters and subsequently scored the alignment with the norMD tool [36]. Since it would be impractical to perform this analysis for each of the 4.1 million CDSs for which an ortholog is found, we selected a representative subset of CDSs, such that: *i*) each chosen CDS has at least one ortholog from each taxonomic level; *ii*) it has no more than 150 orthologs in total, in order to keep multiple alignment computation times reasonable; and *iii*) all orthologous groups are disjoint (each CDS from any group belongs to this group only), in order to avoid sampling bias. There are 2,384 such CDSs with a total of 190,187 orthologs distributed across the taxonomic levels as follows: 3.3% at species level, 8.6% at genus, 3.9% at family, 11.3% at order, 12.5% at class, 30.7% at phylum, and 29.8% at domain level.

**Figure 5:**
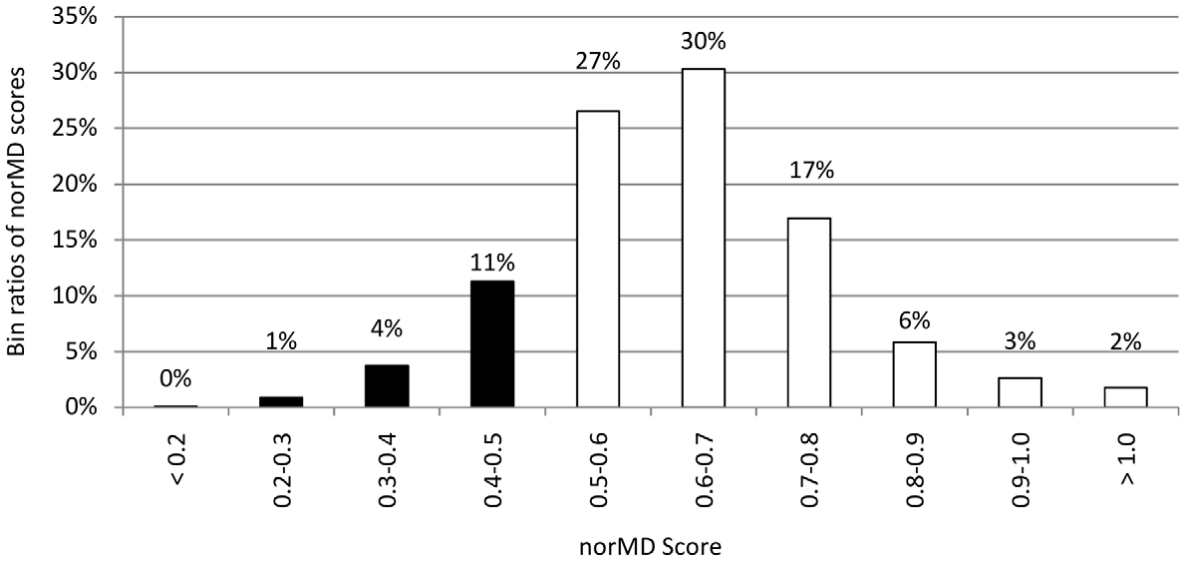
Distribution of norMD scores for the 2,384 multiple alignments. To investigate the specificity of xBASE-Orth we selected 2,384 CDSs such that: *i*) each CDS has at least one ortholog at each taxonomic level; *ii*) it has no more than 150 orthologs in total; and *iii*) the ortholog groups (OGs) for each of the 2,384 CDSs are disjoint. For each of the CDSs, we fetched the orthologs predicted by xBASE-Orth, performed multiple alignment of the sequences with ClustalW2, and scored the alignment with the norMD tool. The x-axis lists the chosen bin ranges for the norMD value. The y-axis depicts the distribution of the observed norMD values across the bins. A value of 0.5 or greater is considered to be the cut-off value for a good multiple alignment, indicating high level of sequence similarity. A vast majority (84%) of the chosen 2,384 OGs exhibit suitable sequence similarity and align well, producing norMD values ≥0.5. It is worth noting that on average about 60% of the orthologs fetched for each of the 2,384 CDSs are at phylum and domain level – i.e. xBASE-Orth exhibits good specificity even at large evolutionary distances.

As shown in Figure 5, for 84% of the orthologs groups the computed norMD value is at least 0.5 (mean norMD = 0.63, σ = 0.16), which is considered to be the cutoff value for distinguishing between good and poor multiple alignment quality [36]; in only 16% of the cases the set of discovered orthologs contains some spurious (or very divergent) orthologs. This result indicates that xBASE-Orth provides suitable specificity even at large evolutionary distances (∼60% of the orthologs for the 2,384 CDSs are at phylum and domain level). For comparison, the tree-based Build_Fam algorithm for constructing the HOGENOM database is shown to outperform OrthoMCL in most of the cases for analyzing 219,951 proteins from 50 bacterial, archaeal and eukaryotic genomes and achieves mean norMD = 0.59, where 74% of the groups have norMD ≥0.5 [10].

### The Bacterial Core Genome

The core genome for a taxonomic group is the collection of CDSs that are present in all of the genomes in this group. However, given the variability of approaches and uncertain accuracy of CDS prediction and annotation across all available bacterial genomes, we adopted a less stringent definition, namely that a CDS had to be present in 90% of the set of bacterial genomes to be termed a highly conserved bacterial ortholog (HCBO). Based on the data in the ortholog groups, we have computed a list of the HCBOs for the domain Bacteria by analyzing all 1,288 bacterial genomes (a significant increase on previous efforts which exploited far fewer genomes).

**Figure 6:**
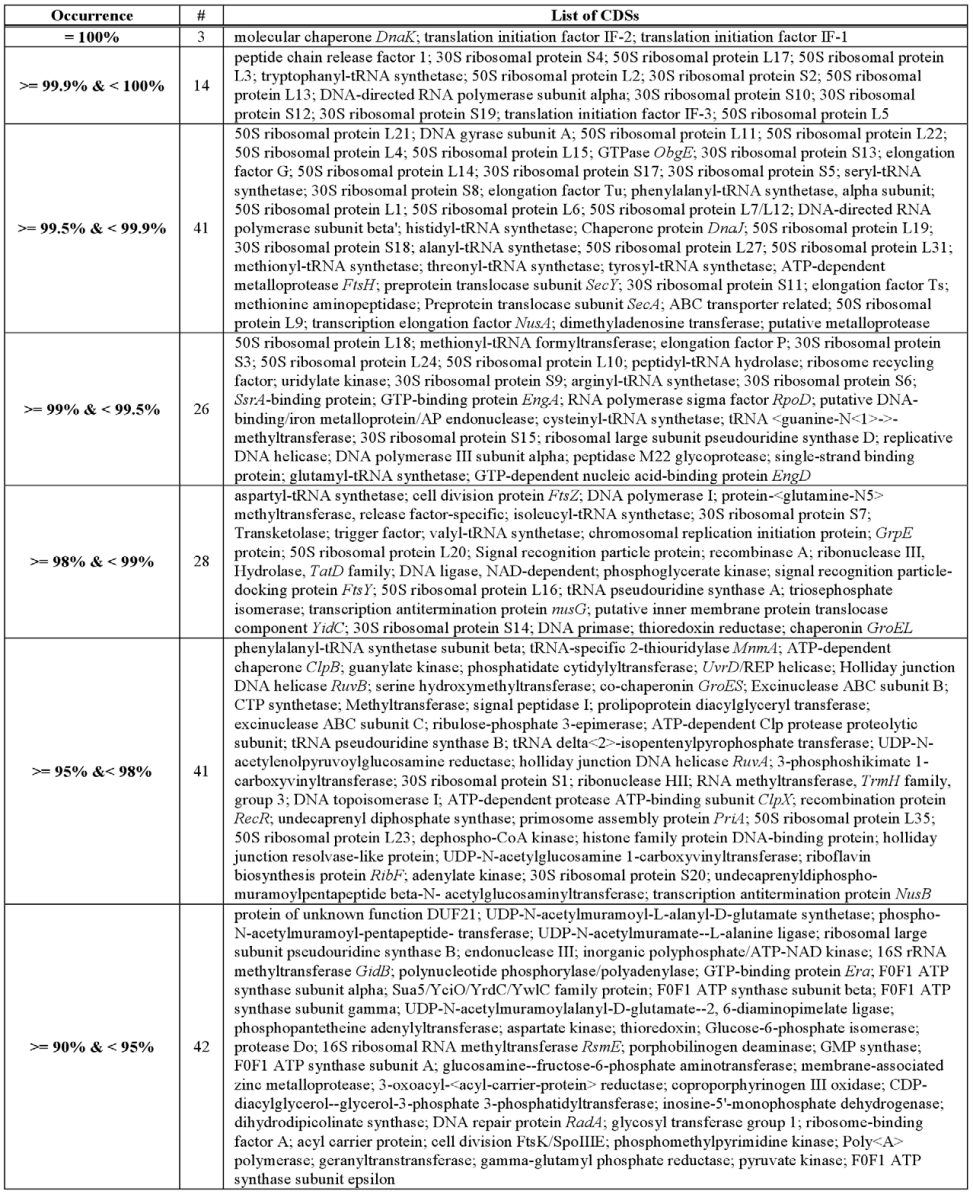
List of potential bacterial core CDSs. For details on potential bacterial core CDSs, see Dataset S2; for details on potential archaeal core CDSs, see Dataset S3.

**Figure 7:**
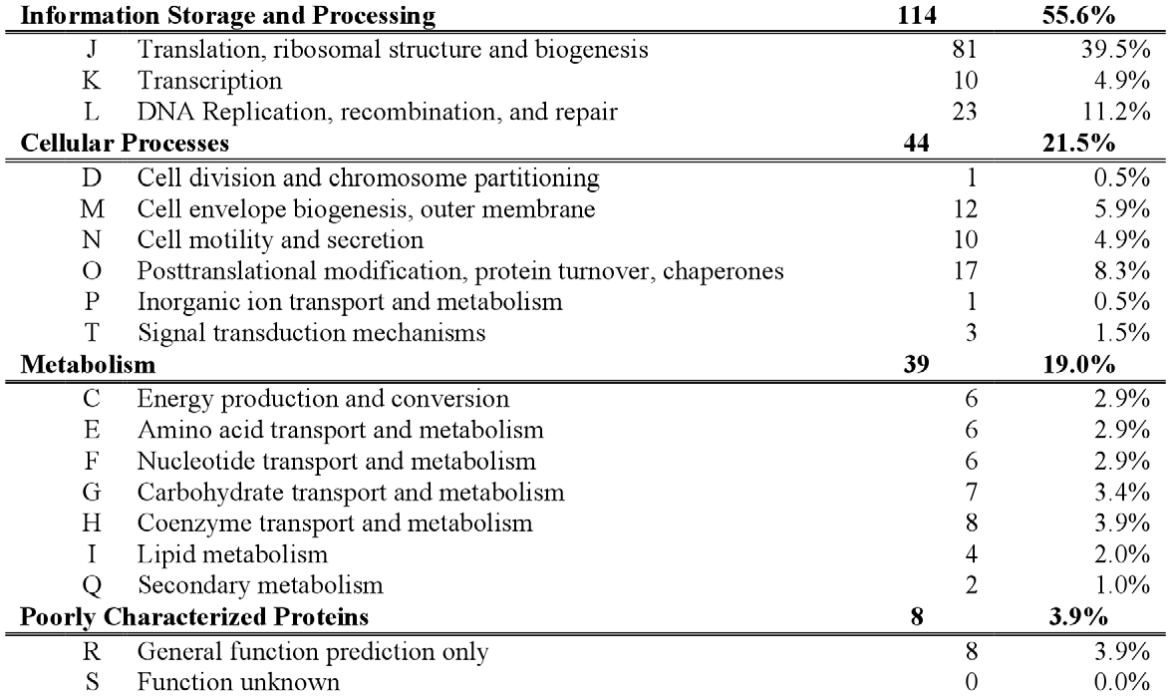
Functional category distribution of HCBOs. We define HCBOs (Highly Conserved Bacterial Orthologs) as CDSs that is present in at least 90% of the considered bacterial genomes. The COG functional assignment was performed using COGnitor.

We identified 195 CDSs as HCBOs in the domain Bacteria (Figure 6, Figure 7 and Dataset S2). The number of HCBOs in the domain Bacteria predicted by xBASE-Orth is in accordance with the estimated ∼250 bacterial extended core CDSs based on the analysis of 573 genomes [29]. Ciccarelli and colleagues [37] analyzed 150 bacterial, 18 archaeal and 23 eukaryotic genomes and determined a set of 36 core CDSs. Our list of HCBOs contains 35 of their 36 core CDSs, but lacks leucyl-tRNA synthetase.

We also computed the archaeal core genome and compared our result with reported data. Previous work predicted archaeal core genome size ranging from 543 CDSs by analyzing 4 genomes [38], 480 by 6 genomes [39], 166 by 41 genomes [40], to 152 CDSs by analyzing and manual curation of the results for almost all of the considered 70 genomes in the unpublished “2009 arCOG update” of [40], available at ftp://ftp.ncbi.nih.gov/pub/wolf/COGs/arCOG/. Our computations produced a list of 261 highly conserved archaeal orthologs (HCAOs) occurring in at least 90% of the 94 genomes (Dataset S3). Overall, our results confirm the findings in the “2009 arCOG update” – 138 of the CDSs were found by both approaches. Only 14 arCOGs were not discovered by xBASE-Orth, possibly due to miscalled CDSs and/or the 24 additional archaeal genomes considered in our analysis. On the other hand, as a result of the more relaxed 90% threshold, xBASE-Orth suggests an additional 123 CDSs that rank as highly conserved archaeal orthologs.

Comparing the COG functional category distributions of the bacterial and archaeal core genomes, it is interesting to note that while the two have similar proportion of core CDSs related to information storage and processing (55.6% in Bacteria vs. 54.5% in Archaea), the archaeal core genome contains more metabolism related CDSs (26.3% vs. 19.0%) and significantly less core CDSs related to cellular processes (7.9% vs. 21.5%).

### Genome Plasticity

The term ‘species pan-genome’ was coined by Tettelin and colleagues [41] and “includes a core genome containing genes present in all strains and a dispensable genome composed of genes absent from one or more strains and genes that are unique to each strain”. Closed pan-genomes indicate species with static genomic content – for such species it is possible to acquire their full CDS repertoire by sequencing enough genomes. In contrast, open pan-genome indicates species with dynamic genomic content, which translates into “infinite” species CDS repertoire - regardless of the number of already analyzed genomes, each newly sequenced strain can be expected to reveal some CDSs unique within species.

**Figure 8:**
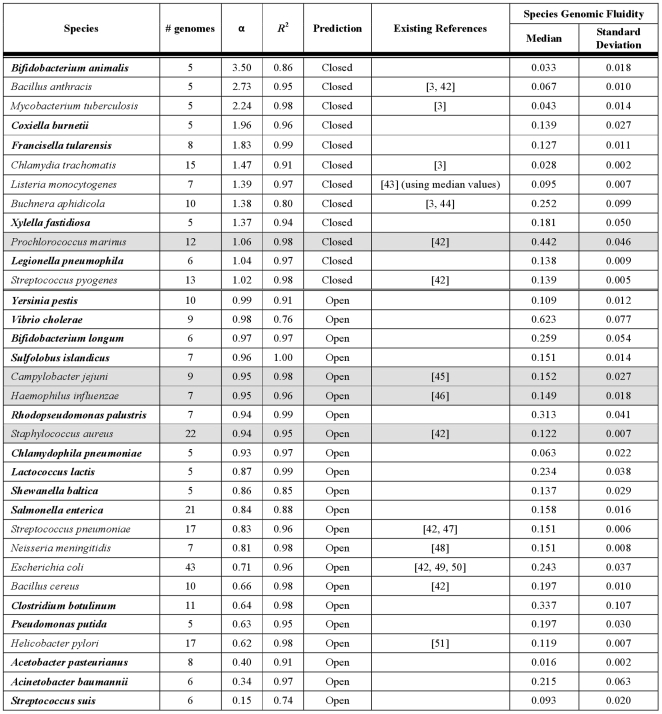
Genome plasticity for 34 species with at least 5 complete genomes. We evaluated the genome plasticity by two alternative methods: evaluation of the pan-genome as proposed by Tettelin *et al.* [42] and computation of the genomic fluidity as proposed by Kislyuk *et al.* [52]. The former approach is based on the assumption that in processing newly sequenced genomes from given species it will become increasingly harder to find novel CDSs and their number *n* grows according to a sub-linear power law *n* = *κ N* ^−*α*^ , where *N* is the number of genomes considered. Species with *α*>1 are said to have “closed” pan-genome, while species with *α*≤1 are said to have “open” pan-genomes. Since the results depend on the order in which genomes are considered, for the *n* values we used medians over 100 random genome order permutations for each species. In most cases the data fitted the model well, with R-squared (goodness-of-fit) values close to 1.0. The latter approach compares each pair of genomes within species to find the proportion of unique/shared CDSs and computes the median species genomic fluidity, where fluidity value of 0.2 implies that 20% of CDSs are unique to their host genomes, while 80% are shared.

**Figure 9:**
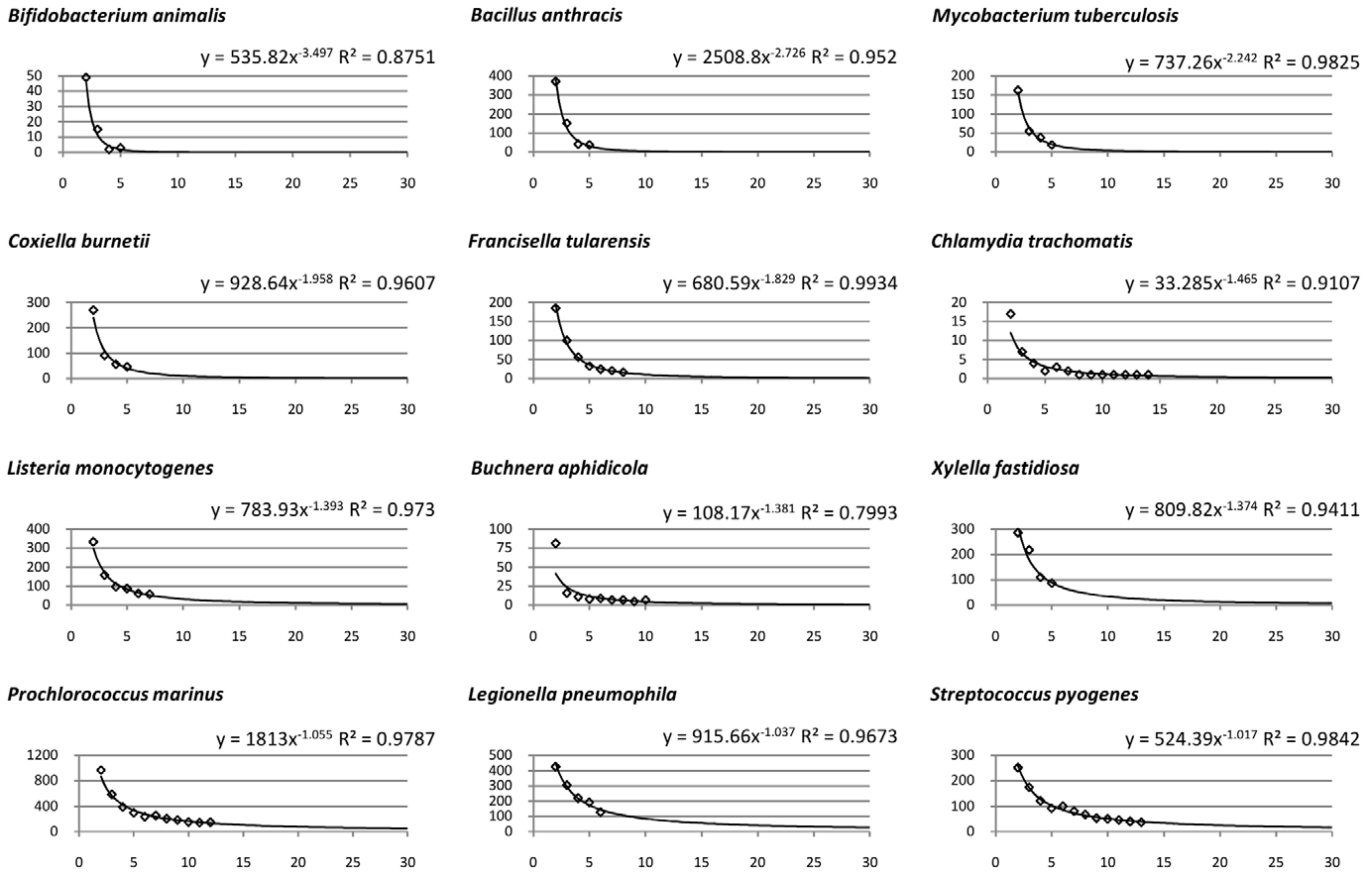
Species with closed pan-genomes. x-axis: number of genomes considered (*N* in the power law model), y-axis: number of new CDSs discovered at each iteration (*n* in the power law model), the curve fitted as power trendline (Excel 2007).

**Figure 10:**
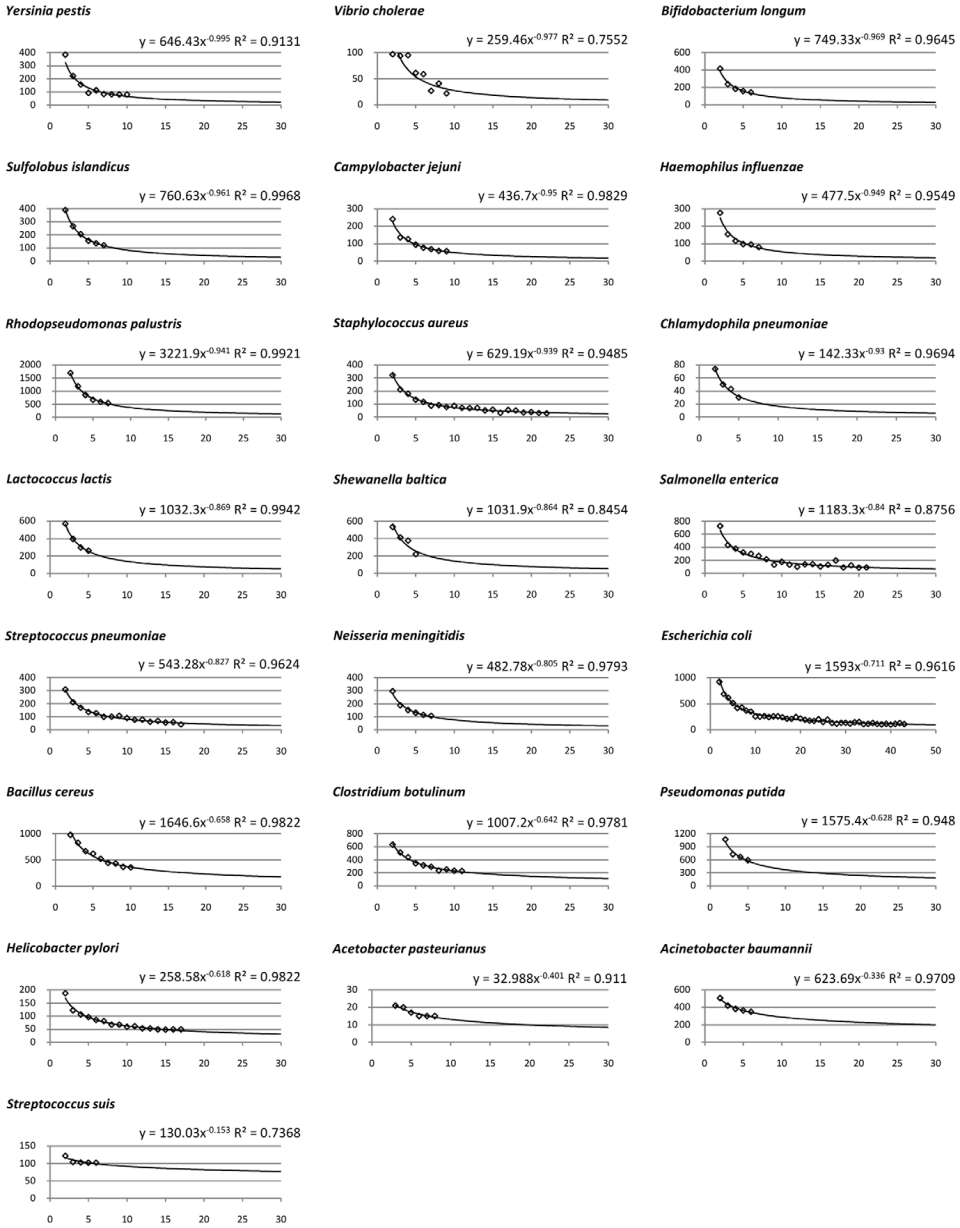
Species with open pan-genomes. x-axis: number of genomes considered (*N* in the power law model), y-axis: number of new CDSs discovered at each iteration (*n* in the power law model), the curve fitted as power trendline (Excel 2007).

We investigated the open/closed pan-genome property for 34 species with 5 or more complete genomes using the technique proposed in [42], defining new CDSs as CDSs with no orthologs (cut-off 70% identity over 70% of the length of the shorter peptide) and no paralogs (90%, 90% cut-offs) and using medians over 100 random genome order permutations. As shown in Figure 8, about one third of considered species have closed pan-genomes (α>1, Figure 9), while the rest have dynamic genomic content (α≤1, Figure 10). We compared our results with previously published predictions available for 15 species [3], [42]–[51] and our predictions generally agree with published data with four exceptions shown in gray in Figure 8. The differences are due to different cut-offs (*S. aureus*), different methodologies in computing the content of the pan-genome (*P. marinus*) and different strains being considered (*H. influenzae*, *C. jejuni*). Particularly interesting is the case of *H. influenzae*. Analyzing only strains isolated in North America, its pan-genome is predicted to be large, but closed [46]. Our analysis is based on 6 of these strains, as well as including the *H. influenzae* 10810 strain isolated in the UK (deposited to NCBI Nov, 2010, accession number FQ312006), which is somehow distinct from the remaining strains – it contains 1,914 CDSs, more than any of the other 6 strains (average of ∼1,700 CDSs) and has more species-unique CDSs (198) than any of the other 6 strains (average of ∼60 CDSs), leading to a borderline open pan-genome prediction. This result illustrates the possible caveat of drawing general conclusions based on closely related isolates (which Hogg and colleagues also pointed out).

We also evaluated the 34 species by computing their genomic fluidity [52]. Results are reported in Figure 8, where a genomic fluidity of 0.2 implies that 20% of the CDSs in a genome pair are unique to their host genome, while 80% are shared. The difference between the two approaches is best illustrated considering *A. pasteurianus* – the analysis of the pan-genome suggests a widely open pan-genome; while according to the genomic fluidity approach *A. pasteurianus* has one of the highest genome similarity, due to the very small proportion of the unique CDSs found for each genome pair.

## Discussion

We have devised an approach that allows for scalable and efficient computation of the bacterial and archaeal ortholog annotations. In addition, due to its hierarchical nature, it is suitable for incorporating novel complete genomes and alternative genome annotations. The proposed xBASE-Orth exploits the available taxonomic information by adopting a “divide and conquer” approach that pragmatically trades accuracy for speed and as a result circumvents the usual high computational cost of ortholog prediction. However, there are several factors that affect the quality of the xBASE-Orth computations – the problem of horizontal gene transfer, quality of the CDS prediction, the approximate nature of our solution, and the sequence similarity paradigm on which it is based, as discussed next.

Our ortholog computation does not take into consideration horizontal gene transfer, which means that for mobile genetic elements such as bacteriophages our approach will predict CDSs to be orthologous when they do not fit the classical Fitch definition of orthologs, i.e. homologous proteins related via speciation [1]. However, this is part of the trade-off between speed and accuracy, as creation and evaluation of phylogenetic trees for each CDS family would be too computationally costly to be feasible. Nonetheless, in almost all cases, xBASE-Orth will provide sound inferences of homology and thereby of function.

Our ortholog computation approach depends on the NCBI's CDS annotation and is therefore affected by the quality of the CDS prediction. The proportion of bacterial genomes (953/1288) in which at least one HCBO was missing based on the CDS annotation is surprisingly high (Dataset S2). For example, among the *Escherichia coli* species, the genome with the highest number of apparently missing HCBOs (ten) is *E. coli* APEC O1. However, performing a search in the *E. coli* APEC O1 genome sequence finds highly plausible hits for all ten HCBOs. To address this problem, we performed a direct six-frame sequence search in all genome sequences which miss at least one HCBO/HCAO using PROMER [53] and a representative subset of the HCBO/HCAO in question as a query set. In about 25% of the cases in which a HCBO cannot be found in the annotated CDSs from a genome, a plausible hit was found through PROMER. The implied defects in annotation underline the need for an optimal and consistent community-wide approach to CDS prediction. Interestingly, performing PROMER searches for apparently missing HCAOs suggest CDS prediction is better in archaeal genomes than in bacteria – plausible hits were found in less than 15% of the cases. These PROMER results have been incorporated into our final assessments of HCBOs and HCAOs presented in Figures 6 and 7 and Datasets S2 and S3.

A comparison between our set of HCBOs (Figure 6) and the expected bacterial core genome, based on what one would predict to be necessary for core informational processes of transcription and translation, reveals some unexpected absences. In some cases, these result from the trade-off between sensitivity and speed. For example, the ribosomal protein S16 is missing from our HCBOs, as xBASE-Orth separates this family of proteins into two clusters – one exclusive to Firmicutes, the other from all other bacteria (present in 76.3% of genomes). Performing a six-frame PROMER alignment reveals only less than 2% of all cross-cluster CDSs pairs have detectable similarity. Being an approximate solution (using pan-genomes rather than the full CDS collection), xBASE-Orth fails to find this similarity, due to selecting a quite distinct CDS as a representative of the S16s in Firmicutes.

Ribosomal protein S21 is also absent from our HCBOs. The S21 proteins are separated into several ortholog groups – the largest cluster, present in 71.5% of genomes, contains CDSs from all phyla except Deferribacteres; two clusters for Cyanobacteria; three clusters for Tenericutes; four clusters for Chloroflexi and eight clusters for Proteobacteria. All S21 proteins in Deferribacteres are grouped together and form the second largest cluster including S21s from some Alphaproteobacteria and Deltaproteobacteria genomes. An attempt to reconcile the two largest clusters using six-frame PROMER alignment reveals no detectable similarity between any pair of CDSs from these two clusters. Incorporation of more sensitive approaches to homology searching might solve this problem, but only at the expense of time and resources.

Another surprise stems from the fact that some CDSs, which are correctly predicted as HCBOs, could not be found in certain genomes, even with six-frame PROMER search. Examples include ribosomal protein L2 (occurrence rate 99.9%) not found in *Streptococcus mutans* UA159, ribosomal protein L6 (occurrence rate 99.8%) not found in *Granulibacter bethesdensis* CGDNIH1, ribosomal protein L11 (occurrence rate 99.8%) not found in *Thermoanaerobacter mathranii* subsp. mathranii str. A3 (although ribosomal L11 methyltransferase is found and annotated in the genome). Potential explanations for these anomalies include errors in the processes of sequencing and assembly, extreme sequence divergence, or a genuine absence of the HCBOs in these genomes.

In conclusion, with xBASE-Orth we have circumvented the usual high computational cost of ortholog prediction by adopting a “divide and conquer” approach that pragmatically trades accuracy for speed. We are confident that this approach will provide scalability for some years to come and that our ortholog dataset and predictions of HCBOs and HCAOs will provide a tool for experimentalists to generate laboratory-testable hypotheses.

## Methods

### Computing Paralogs

The computation of the paralogous CDS pairs in each genome is done using the PROMER tool [53] to perform six-frame alignments in amino-acid space of all-vs-all CDSs in a genome, with no explicit identity and coverage thresholds imposed. For each discovered paralogous pair, only the highest scoring alignment is recorded in our database.

### Computing Species Orthologs

A recent study [28] concluded that for relatively close genomes, the BBH (bidirectional best-hit, also referred to as reciprocal best-hit, RBH) approach provides an acceptable trade-off between accuracy and computational time. Our species ortholog computation expands the traditional BBH approach by being “synteny-aware”. For each possible genome pair within a species we perform six-frame whole-genome pair-wise alignments in amino-acid space using the PROMER tool, chosen over BLAST [54] for its computational efficiency and its suitability for closely related genomes. Next, the aligned regions are sorted based on their length. Starting from the longest alignment, orthologs are detected by a PROMER computation of the similarity between CDSs in the aligned regions and selecting the bidirectional best hits (identity ≥70%, coverage ≥70%). A BBH pair is predicted to be orthologous if none of the two CDSs in the pair is part of previously predicted ortholog pair. In this way, we are filtering out a subset of co-orthologs which do not belong to syntenic blocks. Computing the species orthologs as described took ∼10 days on our server and proceeding in the same accurate but computationally intensive manner would require more than 10 years. The vast majority of the time at this step was spent performing alignments and to reduce their number at higher taxonomic levels, we adopted the pan-genome based scheme that trades accuracy for speed.

### Computing Species Pan-Genomes

Starting with the set of all CDSs from a species, we compute all homologous groups (paralogy cut-off: identity ≥90%, coverage ≥90%, orthology cut-off: identity ≥70%, coverage ≥70%) using the single-linkage approach (i.e., a CDS is clustered to a group if it forms a pair with at least one CDS from this group). In the pan-genome we record all CDSs for which no paralog or ortholog is found, as well as a single representative of each homologous group. We chose to use as a representative the CDS with the largest number of orthologs at this level. Note that our pan-genome computation does not depend on the order in which the genomes are considered (a difference with the traditional sequential inclusion pan-genome computation [41]) – starting with and using all CDSs results in more biologically representative and ∼2% smaller species pan-genomes. While this difference is smaller at species level, it grows more substantial at higher taxonomic levels.

### Computing Genus and Higher Level Orthologs

The synteny-aware BBH approach we used for computing species orthologs is not suitable for ortholog prediction in more phylogenetically distant genomes [11]. We compute orthologs at genus and higher levels as follows. First, the pan-genomes of all species in a genus is computed, as described above. Next, the collection of the species pan-genomes is given as input to OrthoMCL, rather than the full genus CDS collection. OrthoMCL performs an all-versus-all protein BLAST of the CDSs, detects the BBH pairs above user-specified match and e-value thresholds and augments the 1∶1 BBH pairs with in-paralogs using bootstrapping and Markov matrices. We refer to the OrthoMCL result as the set of “explicit” ortholog pairs. At the last step in our algorithm, the set of explicit orthologs are expanded by mapping them to each inter-species pair of genomes using the already computed species ortholog and genome paralog data. For example, if CDS X from species A forms an explicit ortholog pair with CDS Y from species B, then each homolog of X in species A forms an “implicit” ortholog pair with each homolog of Y in species B and all such implicit pairs are added to the set of ortholog pairs. The orthologs at family, order, class, phylum, and domain levels are computed in similar manner, adjusting the OrthoMCL parameter values accordingly (min peptide length = 33 aa, max percentage of stop codons = 2%, e-value cut-off = 1×10^−5^, I = 1.5, percent match cut-off = 65% at genus level, 60% family, 55% order, 50% class, 45% phylum and 40% at domain level).

### Computing Genus and Higher Level Pan-Genomes

Computing the pan-genomes at genus and higher levels is done in a manner similar to the one for species pan-genomes. Starting with the collection of pan-genomes of all species within a genus, replace each *explicit* orthologous group with a single representative CDS - the one with the largest number of genus orthologs (both explicit and implicit). Note that we do not consider the implicit genus orthologs data in pruning the genus pan-genome - although it will result in significantly smaller pan-genomes, it will negatively affect the accuracy of the computation for higher taxonomic levels.

## Supporting Information

Dataset S1 **List of the complete prokaryotic genomes used in the analysis.**
(XLS)

Dataset S2 **List of highly conserved bacterial orthologs (HCBOs).**
(XLS)

Dataset S3 **List of highly conserved archaeal orthologs (HCAOs).**
(XLS)
